# Soft Tissue Coverage of the Lower Limb following Oncological Surgery

**DOI:** 10.3389/fonc.2015.00303

**Published:** 2016-01-13

**Authors:** Christine Radtke, Martin Panzica, Khaled Dastagir, Christian Krettek, Peter M. Vogt

**Affiliations:** ^1^Department of Plastic, Aesthetic, Hand and Reconstructive Surgery, Hannover Medical School, Hannover, Germany; ^2^Department of Trauma Surgery, Hannover Medical School, Hannover, Germany

**Keywords:** soft tissue sarcoma, bone sarcoma, limb-sparing surgery, modular endoprosthetic implantation, soft tissue reconstruction, microvascular flap

## Abstract

The treatment of lower limb tumors has been shifted by advancements in adjuvant treatment protocols and microsurgical reconstruction from limb amputation to limb salvage. Standard approaches include oncological surgery by a multidisciplinary team in terms of limb sparing followed by soft tissue reconstruction and adjuvant therapy when indicated. For the development of a comprehensive surgical plan, the identity of the tumor should first be determined by histology after biopsy. Then the surgical goal and comprehensive treatment concept should be developed by a multidisciplinary tumor board and combined with soft tissue reconstruction. In this article, plastic surgical reconstruction options for soft coverage of the lower extremity following oncological surgery will be described along with the five clinical cases.

## Introduction

Soft tissue tumors and bone sarcomas are a heterogeneous class of mesenchymal tumors comprising <1% of all malignancies in adults and represent 15% of pediatric malignancies^1^ (1). The overall mortality rate for soft and bone sarcomas was estimated as 30 and 45%, respectively^1^ (1). Two-thirds of the tumors are located in limbs, most frequently in the lower extremity (46%) (1). Osteosarcoma has been the primary model to base treatment of all sarcomas. Multi-agent chemotherapy regimens have demonstrated an increase in overall survival rates (15–20%) as compared to surgery alone in the 1970s, but more recently, survival has increased to 55–80%^1^ (2).

There has been considerable progress in the management of limb sarcomas over the past few decades. Several decades ago, there was a high rate of limb amputations (38–47%) associated with sarcoma, likely the result of reduced radiotherapy and reconstructive methods (3). The introduction of radiotherapy has considerably improved outcome and in combination with oncological and advanced reconstructive surgery important advances have been made in tumor control and functional limb preservation^1^ (4).

In high-grade malignancies or tumors of borderline resectability, preoperative chemotherapeutical downsizing could be indicated. In the case of non-resectable tumors, especially sarcomas in close proximity of functional structures, isolated limb perfusion can be considered (5). Postoperatively, necessary chemotherapy can be combined with deep wave hyperthermia (6). Although limb amputation may be unavoidable in some circumstances, the combination of limb-sparing and reconstructive surgery can optimize function of the affected limb and avoid the significant psychological impact associated with amputation^1^. Endoprosthetic procedures for skeletal reconstruction have improved functional outcome (7). Currently, 90–95% of limb sarcoma patients may undergo successful limb-sparing procedures with soft tissue coverage when treated at a major center specializing in musculoskeletal oncology (4). Thus, for the majority of soft tissue malignancies and bone sarcomas of the limb, limb-sparing surgery performed in an interdisciplinary team (8) is an important treatment option.

## Surgical Planning and Decision-Making Considerations

A meticulous surgical technique is crucial to ensure an optimal oncological and functional outcome for the patient. Successful limb-sparing surgery consists of three interdependent stages performed in sequence as follows:

1. Tumor resection with appropriate oncological margins,
2. Reconstruction and stabilization of the involved bone and joints, and
3. Restoration of the soft tissue envelope and restoration of function.

The overall aim of oncological surgery followed by soft tissue reconstruction is to carry out a wide compartmental excision for maximal tumor removal, yet to preserve limb function. The excision is defined as wide when the distance between the histologically defined tumor and the excision margins are at least 2 cm (3). However, if there is an anatomical barrier such as deep or muscle fascia that is intact, which separates the tumor from the excision border, the tumor may be considered wide with an excision distance <2 cm (3). The European Society for Medical Oncology (ESMO) guideline does not state a specific margin size. However, it does recommend that radiation therapy can be used for tumors larger than 5 cm^2^^,^^3^. Because tumor excision often leads to potentially large tissue defects, including bone, joint, and tendon exposure, reconstructive surgery is an important and critical element (9, 10). For skeletal and joint reconstruction, advances in commercial modular endoprosthetic devices have importantly advanced the field^1^ (7, 11). With the development of modular endoprosthetic devices with their large range in size and adaptability, the surgeon can focus on optimizing the oncological resection procedure, having the knowledge that appropriate prosthetic components will likely be available even if the surgical procedure needs to deviate from the preoperative plan. Thus, modern modular endoprosthetic reconstruction plays an important role in limb-sparing surgery for bone sarcoma resection. Ongoing work to develop better approaches for attachment of tendon to endoprosthetic devices such as novel clamps and in growth-promoting surfaces to promote may lead to improved junctional strength^1^ (11). The most common site for primary bone sarcomas is the distal femur. Endoprosthetic reconstruction of this region is of particular challenge because the cruciate and collateral ligaments must be removed thus reducing stability^1^ (12). Appropriate soft tissue coverage is imperative to decrease the risk of secondary periprosthetic infection.

After tumor resection of the lower extremity complex, defects are anticipated and multiple variables must be considered for soft tissue reconstruction. A large number of details must be taken into consideration when planning a reconstruction, especially after oncological surgery. One must consider the timing of reconstruction, size and location of the defect, involvement of neurovascular structures, and exposure or resection of bone, tendons, and nerves. Donor site morbidity, disease prognosis, and the patient’s previous level of function and expectations of restored function must be evaluated as well.

The reconstructive ladder as a concept for wound closure has gone through several adjustments over time (13–15). While the concept of using the simplest approach and moving up the ladder to more complex approaches is certainly important, there may be times with oncological defect surgery where this approach may not be valid (13).

This led to the idea of the “reconstructive elevator,” which was introduced by Gottlieb and Krieger (16). While still admitting to the idea of increasing levels of complex difficulty, the “reconstructive elevator” offers the flexibility to elevate directly to an appropriate level of complexity as necessary (17). This concept draws attention to the importance of selecting the most appropriate level of reconstruction instead of selecting the least complex that is often the case in soft issue coverage after oncological surgery.

## Determining the Optimal Timing for Soft Tissue Reconstruction

It was demonstrated with regard to traumatic wound coverage of the lower limb that microvascular tissue transfer after 5–21 days post-trauma resulted in higher flap failure rates and wound infections (18). In soft tissue coverage following oncological surgery, an early time point for wound closure is preferred, but multiple stage/sequential procedure might be necessary for the achievement of a R0 resection and temporary closure is applicable. However, when there is R1 or R2 status and chemo- and radiation therapy is required, stable wound closure is essential before the onset of these therapeutic regimens.

There are several advantages for immediate reconstruction to be carried out at the time of tumor resection. One is that the anatomical perspective of the oncological defect can be assessed prior to scar formation. This will minimize surgical dissection of, for example, exposure of vessels for microvascular repair that would be necessitated if there was a delay with scar formation (19, 20). Another advantage is the psychological benefit to the patient. However, reconstruction is delayed in cases where the margins of the resection site are not clear or when the patient has issues with would healing (20).

## Soft Tissue Reconstruction

Plastic surgical reconstruction options for soft coverage of the lower extremity following oncological surgery will be described by means of five clinical cases.

### Case 1

**Figure 1:**
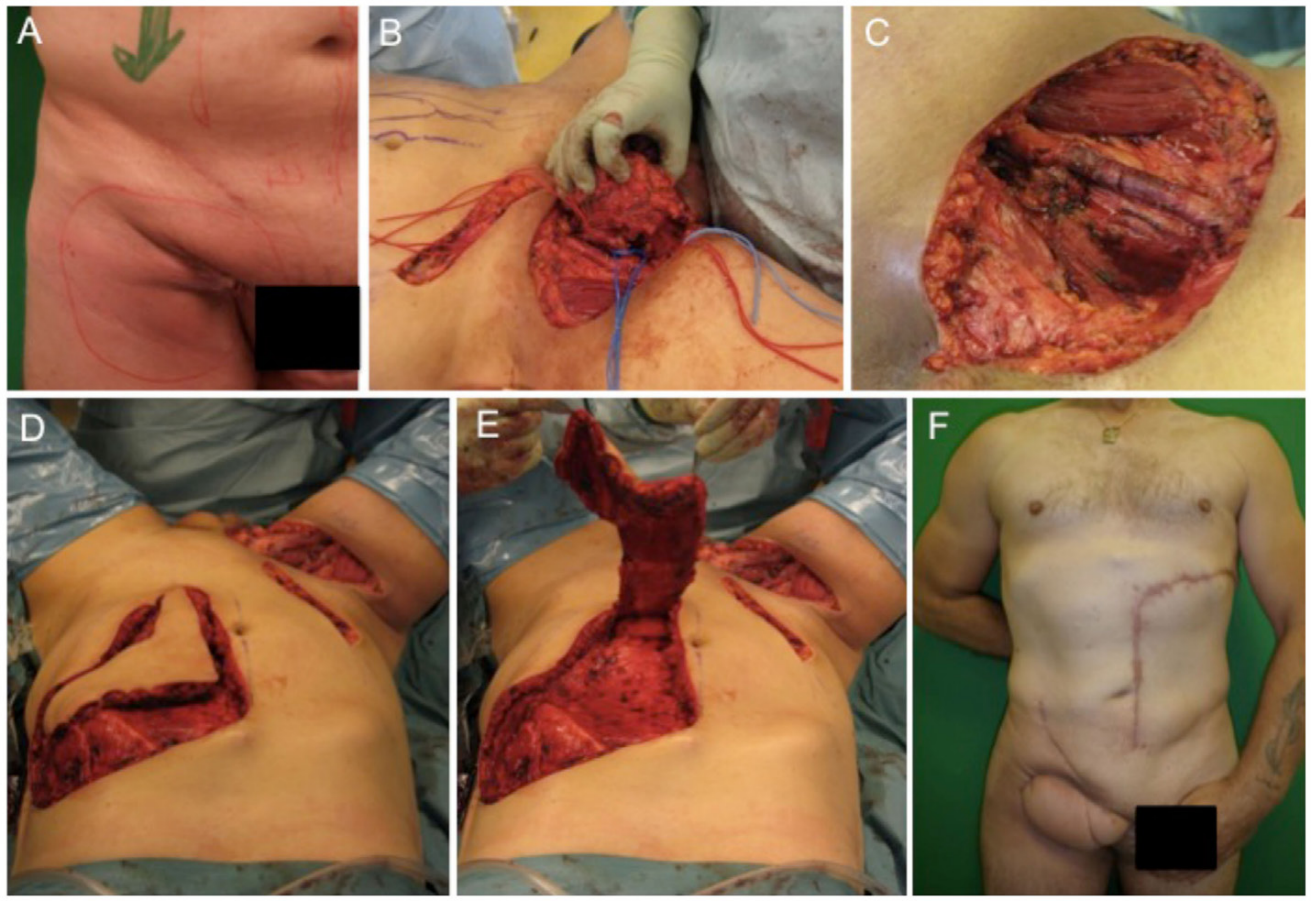
**Demonstration of tumor at the right inguinal region expanding on the proximal thigh (A), complete metastatic resection was performed with preservation of inguinal vessels and femoral nerve (B,C)**. As a next step, an extended VRAM flap from the contralateral side was prepared and transferred for defect closure **(D,E)**. Long-term results revealed complete removal and stable coverage with minimal donor morbidity **(F)**.

The first case describes the soft tissue coverage in the proximal thigh/inguinal region. A 55-year-old male patient presented with an ulcerating metachronous metastasis in the right inguinal region (Figure 1A) after resection of a squamous cell carcinoma (SCC) to the anus limited to the anal margin only infiltrating the perianal skin and without invasion of the sphincter muscle, which was resected 11 months before (pT1, pN0, pM0, R0). The surgical excision of the metachronous metastasis resulted in a soft tissue defect with an extension of 11 cm × 11 cm as illustrated in Figures 1B,C. Here, the complete resection with preservation of vessels and femoral nerve followed by soft tissue coverage with an extended vertical rectus abdominis myocutaneous (VRAM) flap (Figures 1C–E). After wound healing, adjuvant chemotherapy with Cisplatin/5-Fluorouracil followed by radiotherapy was performed according to the anal cancer treatment protocols for metastatic diseases following current guidelines (21). Long-term follow-up demonstrated stable coverage (Figure 1F). This case represents an individualized tumor treatment concept and a challenging situation for soft tissue coverage because of its large soft tissue defect in the groin with the need of a soon adjuvant therapy.

Treatment options for the groin and thigh reconstruction include as local flaps include sartorius, the tensor fascia latae, or the rectus femoris flaps. With regard to tumor size in the presented case, an extended VRAM flap was performed.

### Case 2

**Figure 2:**
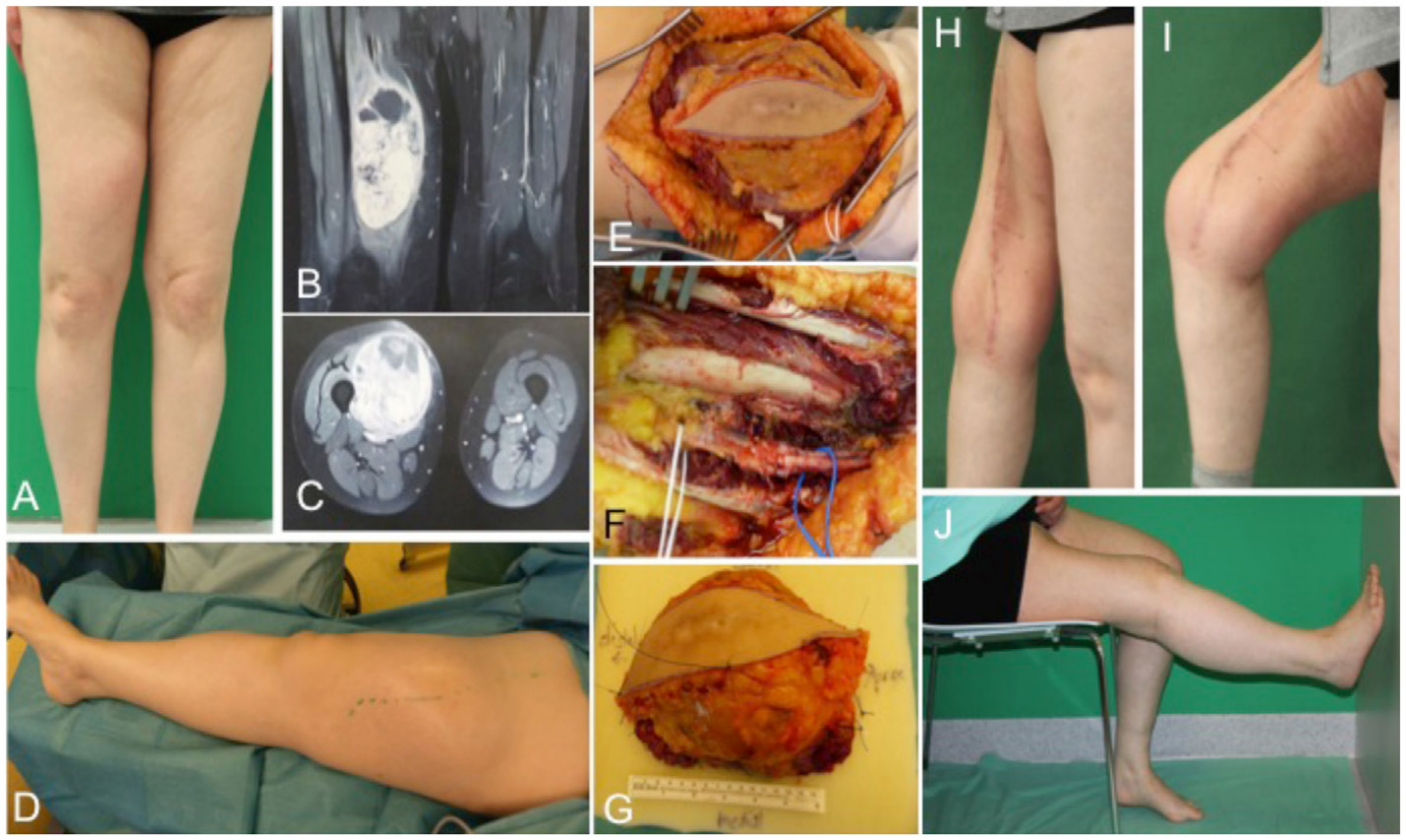
**Preoperative clinical presentation of a 54-year-old female with an undifferentiated myxofibrosarcoma G3 in the anterior compartment of ventral thigh and correlation with MRI (A–C)**. Intraoperative situs **(C–F)** with complete tumor removal **(G)**. Long-term results **(H–J)** of primary closure showing the esthetic outcome and maintained function of the right limb.

A 54-year-old female patient presented with a gradually growing non-inflammatory and indolent tumor of the right thigh (Figures 2A,D). Magnetic resonance imaging (MRI) revealed a heterogeneous tumor involving the vastus lateralis, medialis, and intermedius muscles (Figures 2B,C). A biopsy confirmed an undifferentiated myxofibrosarcoma. CT scans showed no evidence of metastasis. The tumor was removed with a complete size of the resected tissue of 15 cm × 8 cm × 7.5 cm under preservation of vessels and the femoral nerve (Figures 2E–G). Primary closure could be performed after radical resection and histology revealed an undifferentiated myxofibrosarcoma pT2b, pN0, pM0, R0; G3 (FNCLCC). Clinical follow-up examination 1 year after surgical treatment showed stable long-term results with a range of motion of right knee for extension/flexion 0/0/120° and 60/0/40° of the right hip joint (Figures 2H–J).

Treatment options for the groin and thigh reconstruction include sartorius, tensor fascia latae, or rectus femoris local flaps (22). With regard to tumor size in the presented case 1, an extended VRAM flap was performed. In the illustrated case 2, the defect could be closed primarily. If primary closure cannot be performed, the use of local muscle is in most cases the best treatment option and the need for free flaps is only in extensive cases necessary. For anastomosis of a microvascular flap, the deep inferior epigastric, the superficial epigastric, the superficial circumflex iliac, or the femoral vessels could serve as recipient vessels where end to side anastomoses should be preferred to preserve distal blood flow (23).

### Case 3

**Figure 3:**
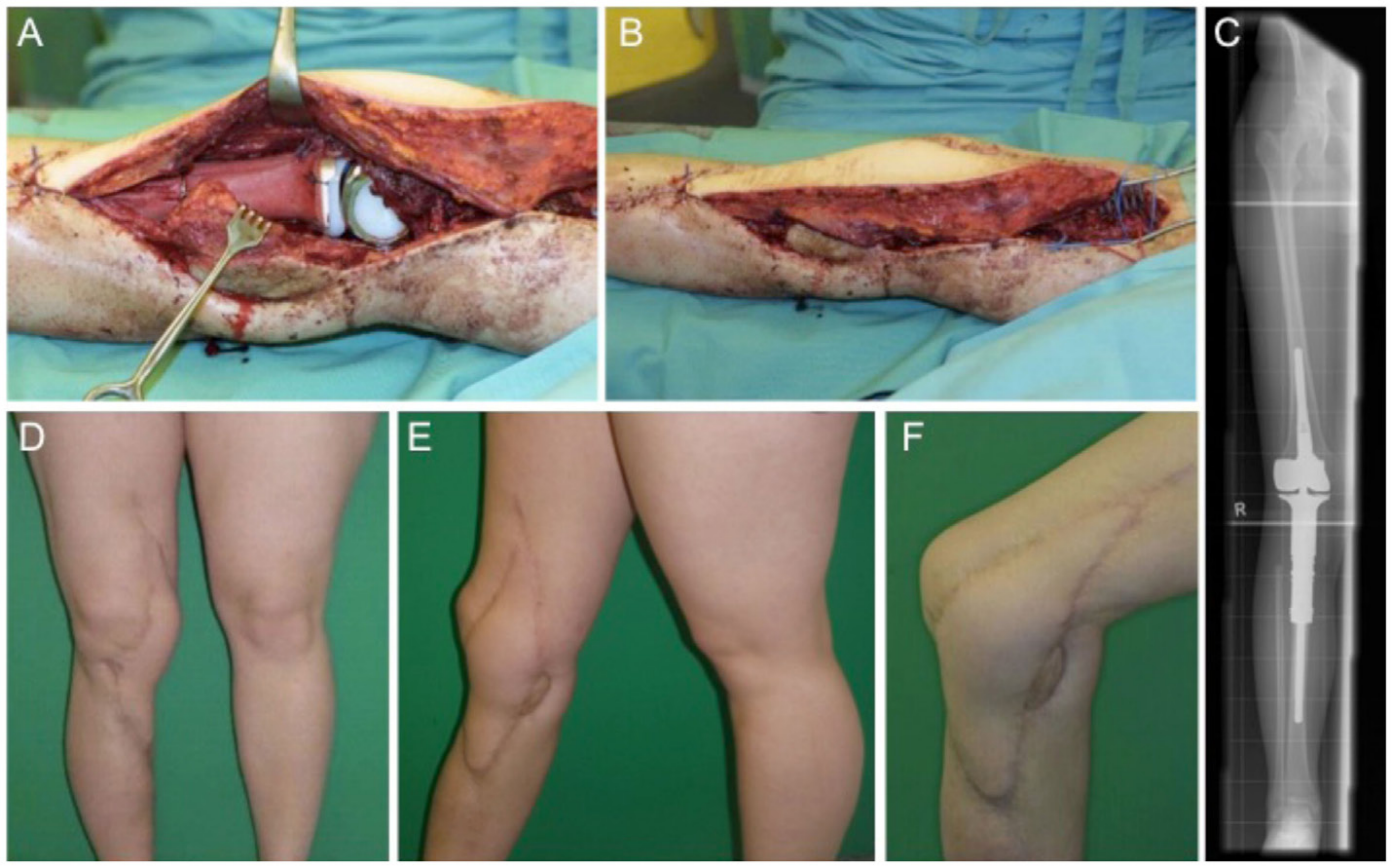
**Intraoperative situs with implanted tumor prosthesis (MUTARS^®^) after extraarticular tumor resection of the knee joint (A,B) and coverage with a microvascular latissimus dorsi flap (B)**. **(C)** shows the corresponding x-ray image with a proximal tibial replacement. Post-operative esthetic and functional outcome **(D–F)**.

This case describes a 15-year-old female with confirmed osteosarcoma of the proximal tibia and treatment with an induction chemotherapy according to the Cooperative German–Austrian–Swiss Osteosarcoma Study Group (COSS) protocol (24, 25). After completing neoadjuvant therapy, the patient was scheduled for extraarticular knee joint resection. Modular endoprosthetic knee reconstruction was performed with a proximal tibia replacement and a knee reconstruction implant (MUTARS^®^) using a trevira tube for soft tissue fixation. Initial soft tissue coverage with a medial gastrocnemius flap failed due to early post-operative infection. After multiple debridement and revision surgery with antibiotic spacer application, infection was treated successfully. A new modular endoprosthetic replacement (Figures 3A–C) was then covered with a microvascular latissimus dorsi flap as limb salvage procedure (Figures 3B,D–F). Histology confirmed complete R0 resection of the osteosarcoma pT2, pN0, pM0; G3. The patient regained good post-operative function without signs of extension gap and long-term stable soft tissue reconstruction (Figures 3D–F).

Local flap for soft tissue coverage at the knee, the gastrocnemius flap is the first choice (26). From the gastrocnemius muscle, either the medial or the lateral or both heads can be transferred for soft tissue coverage. Usually, the medial head is larger in comparison to the lateral gastrocnemius head. Other possibilities besides free flaps include a reversed anterior lateral thigh (ALT) flap or a reverse vastus lateralis flap. In flap decision-making for knee reconstruction, the range of motion of the knee as highly mobile joint has to be taken into consideration and the amount of necessary surface area has to be calculated carefully within the flap design (23).

### Case 4

**Figure 4:**
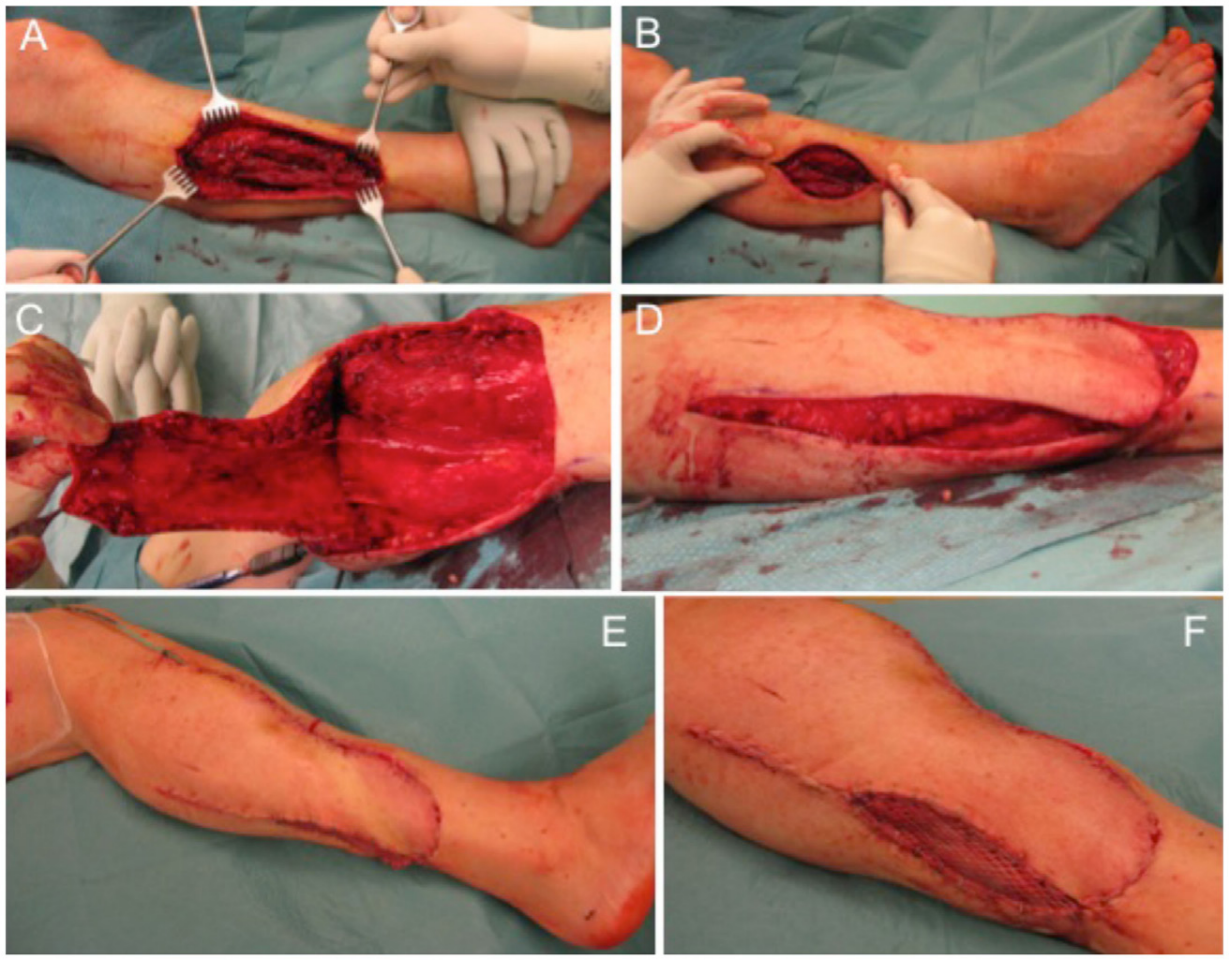
**Defect of the lower leg after tumor removal (A) with remaining defect at the lateral side (B)**. A fasciocutaneous transposition flap is raised **(C)** and transferred ventrally into the defect **(D)**. Full coverage can be achieved **(E)** and remaining areas at the donor side can be transplanted with split thickness skin graft **(F)**.

The next case describes a 51-year-old female patient with a gradually growing non-inflammatory and indolent swelling of her right lower leg. MRI showed a non-homogeneous tumor in the antero-lateral compartment of right leg, within the tibialis anterior and the extensor digitorum longus muscles. CT scans of her abdomen and chest and other studies showed no evidence of metastasis. Biopsy confirmed the diagnosis of a sarcoma. Subsequently, complete tumor removal was performed (Figure 4A) leading to a soft tissue defect of 10 cm × 7 cm (Figure 4B). Wound closure was performed with a fasciocutaneous transposition combined with a small split thickness skin graft at the donor side (Figures 4C–F). Histologically, the tumor was graded as pleomorphic undifferentiated sarcoma pT1, pN0, pM0, R0; G3. After wound healing, adjuvant radiotherapy with total dose of 56 Gy was conducted. Clinical follow-up 1 year after surgical treatment showed a stable complete soft tissue coverage with a range of motion for ankle dorsiflexion/plantar flexion of 5/0/30° and for eversion/inversion of 5/0/20° on the operated right side in comparison to the unaffected left side for ankle dorsiflexion/plantar flexion with 15/0/30° and eversion/inversion 10/0/20°.

Reconstruction options for the lower leg with regard to local flaps are limited particularly for the lower third can be challenging, thus free flaps are often required. Here, we performed as a prerequisite an angiography. Underlying stenosis of the arteries can often be diagnosed and the lower limb revascularization preceding surgical wound coverage is necessary to reduce complication rates. An algorithm was described to improve the success of microvascular tissue transfer on the lower extremity (27).

### Case 5

**Figure 5:**
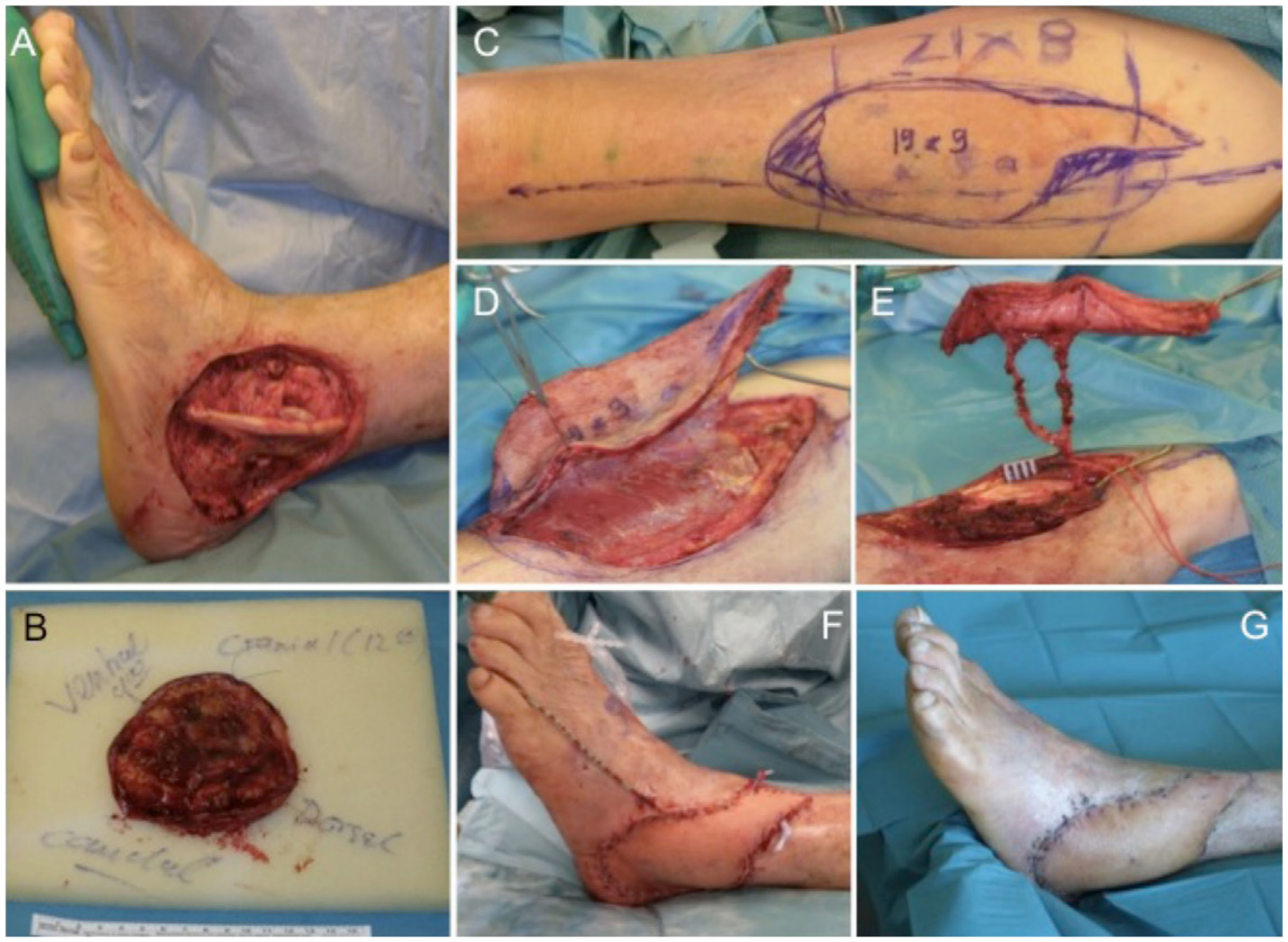
**Extensive soft tissue defect at the lateral malleolus (A) after tumor excision (B)**. For wound coverage, an ALT flap is prepared **(C,D)** with a long pedicle **(E)** for microvascular anastomosis. The ALT flap engrafted into the defect shows intraoperatively **(F)** and after wound healing a very satisfying result **(G)**.

An 81-year-old male patient presented with a previously incomplete (R1) resected primary SCC on the lateral aspect of his left ankle. X-ray examination revealed no involvement of the underlying bone and CT scans ruled out any further metastatic involvement. Tumor resection of the SCC resulted in an extended soft tissue defect (Figures 5A,B) that required microvascular flap coverage and preparation of an ipsilateral ALT flap with a size of 19 cm × 9 cm was performed (Figures 5C–E). Histology confirmed complete tumor removal of the SCC (pTx, pN0, pM0, R0; G2). Complete wound closure could be achieved (Figure 5F) with long-term functional and esthetic outcome (Figure 5G).

Possibilities for local flaps at the foot include the reversed sural, dorsalis pedis, and abductor digiti minimi flaps. For free-flap anastomosis, recipient vessels are the anterior, posterior, peroneal, and dorsalis pedis artery with concomitant veins. The plantar surface requires a separate approach of exposure to high pressure during walking and mechanical stress. Here, an instep flap could be used as an option (22).

## Post-Operative Care and Immobilization

In the post-operative phase, the limb should be maximally elevated for swelling reduction that could potentially compromise the flap used for soft tissue coverage^1^ (22). It is critical that the limb is immobilized and sterile dressing applied to maximize tissue survival (22, 28). The 24-h post-operative period is critical because a high incidence of complication related to micro-revascularization of the flap occurs (22, 28). Hematoma formation must be reduced and large-bore closed suction drains are helpful in this regard^1^ (22). In case of hematoma development immediate and aggressive treatment in the operating room should be carried out to prevent occlusion/compression of microvascular anastomoses, which could lead to perfusion complications of the flap as an immediate effect and the secondary effect of infection, especially in case of implanted endoprosthesis^1^ (22). Subsequent wound care, physical therapy, and potential tumor adjuvant therapy are essential to complete the therapeutic process (28). Completion of the therapy should include standard wound care and physical therapy with the potential for adjuvant therapy for tumor treatment (22, 28).

## Conclusion

Limb salvage in patients with sarcoma is possible with an acceptable outcome by selective combination of required treatment modalities. Currently, primary amputation is usually only performed in cases where the tumor infiltrates major neurovascular structures, bone or joint and when not even marginal resection is feasible. In these cases, the great risk of local recurrence or of poor limb function favored amputation. Clearly, it is important that patients should be provided with solutions that address improvement in function, but cosmetic and psychological issues should be addressed as well. For patients initially thought to have unsalvageable limbs because of tumor size and location, reassessment after preoperative chemotherapy may allow reconsideration of limb-sparing procedures. Therefore, a careful re-evaluation of the patient following adjuvant treatment is necessary for defining a meticulous multidisciplinary surgical plan. Limb-sparing procedures combined with soft tissue coverage after oncological surgery should not be limited to patients with a curative goal, patients in a palliative stage of disease can benefit from surgery in terms of pain reduction and improvement of quality of life. Finally, given the complexity of a multidisciplinary approach, individualized treatment should be performed in major centers specializing in musculoskeletal oncology.

## Conflict of Interest Statement

The authors declare that the research was conducted in the absence of any commercial or financial relationships that could be construed as a potential conflict of interest.

## Footnotes

^1^www.sarcoma.org

^2^www.esmo.org

^3^www.current-oncology.com
